# Respiratory and Cardiovascular Activity of LENART01, an Analgesic Dermorphin–Ranatensin Hybrid Peptide, in Anesthetized Rats

**DOI:** 10.3390/ijms26157188

**Published:** 2025-07-25

**Authors:** Piotr Wojciechowski, Dominika Zając, Adrian Górski, Wojciech Kamysz, Patrycja Kleczkowska, Katarzyna Kaczyńska

**Affiliations:** 1 Department of Respiration Physiology, Mossakowski Medical Research Institute, Polish Academy of Sciences, Pawińskiego 5, 02-106 Warsaw, Poland; pwojciechowski@imdik.pan.pl (P.W.); dzajac@imdik.pan.pl (D.Z.); 2 Department of Inorganic Chemistry, Faculty of Pharmacy, Medical University of Gdansk, 80-416 Gdansk, Poland; adrian.gorski@gumed.edu.pl (A.G.); wojciech.kamysz@gumed.edu.pl (W.K.); 3 Department of Biomedical Research, National Medicines Institute, Chełmska 30/34, 00-725 Warsaw, Poland; hazufiel@wp.pl; 4 Maria Sklodowska-Curie Medical Academy in Warsaw, Pl. Żelaznej Bramy 10, 03-411 Warsaw, Poland

**Keywords:** ranatensin, dermorphin, respiratory effects, apnea, cardiovascular effects

## Abstract

Opioids are among the most effective drugs for treating moderate to severe pain. Unfortunately, opioid use, even short-term, can lead to addiction, tolerance, overdose, and respiratory depression. Therefore, efforts to design and develop novel compounds that would retain analgesic activity while reducing side effects continue unabated. The present study was designed to investigate the respiratory and cardiovascular effects of the hybrid peptide LENART01, which has evidenced potent antinociceptive and antimicrobial activity. This hybrid peptide, composed of N-terminally located dermorphin and C-terminal modified ranatensin pharmacophore, was tested in vivo in anesthetized rats. The main effect of LENART01 was apnea in 70% of examined animals, sighing, and a significant increase in blood pressure. Interestingly, the hybrid induced sighs less frequently than ranatensin, and apnea dependent on vagus nerve mu opioid receptor activation much less frequently and less intensely than dermorphin itself. This shows that LENART01 is a safer opioid system-related agent as compared to dermorphin for its prospective use in the treatment of pain.

## 1. Introduction

The development of effective pain treatments remains a major clinical priority, particularly in the context of the opioid crisis. While opioids such as morphine are highly effective analgesics, their use is severely limited by serious side effects, including tolerance, dependence, respiratory depression, and overdose-related fatalities [1,2]. In response, substantial efforts have been devoted to designing safer analgesics—one promising strategy involves the creation of multifunctional or hybrid molecules that combine two distinct pharmacophores within a single structure [3]. In fact, hybrid molecules merge two or more pharmacophores into a single compound [4,5,6], which results in targeting multiple receptor systems simultaneously, potentially enhancing therapeutic outcomes and reducing side effects.

LENART01 is a rationally designed hybrid peptide constructed from two distinct pharmacophores: dermorphin—a heptapeptide derived from the skin of the Amazonian frog Phyllomedusa sauvagei known for its potent μ-opioid receptor (MOR) agonism and strong significant analgesic effects [7,8], and ranatensin—an undecapeptide isolated from the northern leopard frog Rana pipiens [9], currently Lithobates pipiens [10], that exhibits activity towards dopamine D2 receptors (D2R) and has been shown to modulate central nociception [11]. Previous studies have confirmed that LENART01 acts as a full agonist at MOR and interacts with D2R in rat brain and spinal cord [3,12]. In vitro binding studies showed that it acts as a potent and complete agonist of human MOR and binds selectively to it over other opioid receptor subtypes [3]. It exhibits antinociceptive [3] and antimicrobial [13] properties and demonstrated a favorable behavioral profile after subcutaneous (s.c.) administration in mice, with less sedation and motor impairments than morphine [3]. Importantly, it does not induce withdrawal syndrome in mice, which may be related to its dopaminergic activity and modulation of D2R expression [3,14].

Although LENART01 has been pharmacologically validated as an analgesic, its impact on respiratory and cardiovascular function remains unknown. Given that dermorphin has been shown to cause apnea and hypotension in anesthetized rats [15,16], and that ranatensin has been associated with conflicting cardiovascular effects (both hypotensive and hypertensive responses) [9], while overall, D2R agonists are associated with cardiovascular (CV) complications including orthostatic hypotension and heart failure [17,18], it is crucial to evaluate whether their combination affects vital autonomic systems.

Therefore, the purpose of this study was to investigate LENART01-induced effects on the respiratory and cardiovascular systems after intravenous (i.v.) administration in rats. To better understand the underlying mechanisms, we compared LENART01’s effects with those of its individual pharmacophores and employed pharmacological antagonists of MOR, D2R, and BB1/BB2 bombesin receptors to elucidate receptor-level contributions. These investigations are essential for defining the safety and systemic effects of LENART01, especially in light of its potential clinical utility as an opioid-based analgesic with an improved side-effect profile.

## 2. Results

### 2.1. LENART01 Dose Response

The chosen dose of 15 μg/kg was determined based on the observed dose-dependent effect of LENART01 on the frequency and duration of apneic events (see Table 1). The lowest dose of 5 μg/kg failed to induce any arrest of breathing, while the highest dose used, 20 μg/kg, appeared to be associated with a markedly increased number of apneas exceeding a duration of 30 s, which had to be terminated by the application of a respiratory pump. Therefore, a lower dose of 15 μg/kg producing a higher number of apneas from which the animals emerged spontaneously was chosen for further study. Regarding the T_E_/T_E baseline_ ratio, a dose-dependent increase was observed, with higher doses of LENART01 generally associated with elevated ratios.

### 2.2. Respiratory Effects of LENART01: The Effects After Blockade of MOR, BB1/BB2, D2R, and After Cervical Vagotomy

Intravenous administration of LENART01 at a dose of 15 μg/kg induced apnea in 10 out of 14 rats, with four instances exceeding a duration of 30 s. The remaining six episodes of apnea exhibited a median (IQR) duration of 11.2 s (10.8). Apnea was not effectively eliminated by blockade of D2 or BB1/BB2 receptors. After pre-treatment with sulpiride, LENART01 evoked apnea in 50% of rats, and after PD176252, in 20%. In cases where apneic episodes were not observed, a decrease in tidal volume (V_T_) was recorded: a 28% reduction in the control LENART01-treated group and reductions of 22% and 18% following pre-treatment with sulpiride and PD176252, respectively. Pre-treatment with naloxone, along with midcervical vagotomy, effectively abolished both apnea and tidal volume response (Table 2).

**Figure 1:**
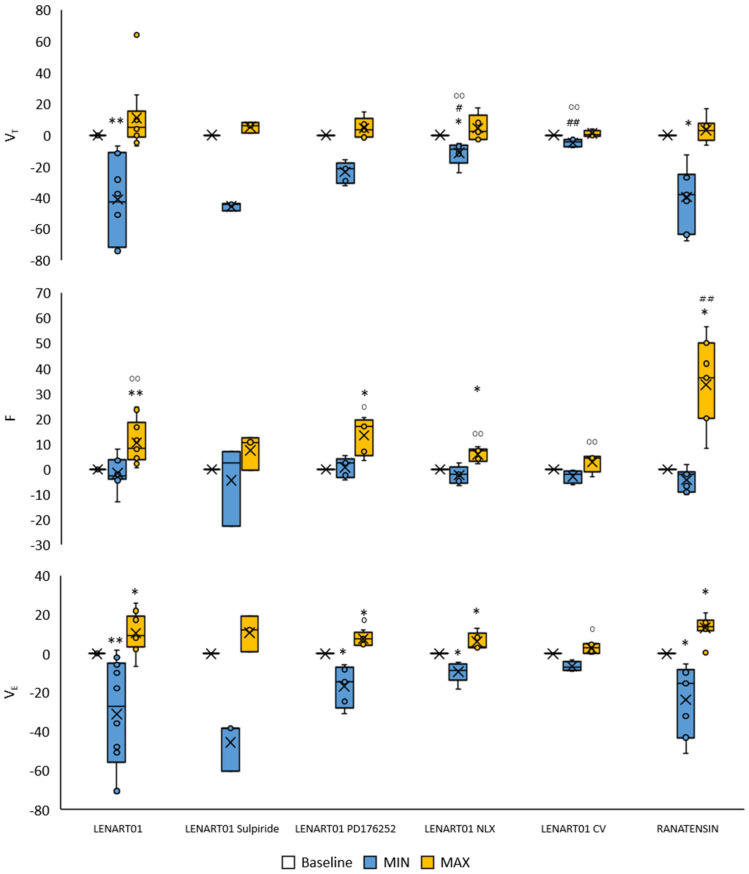
Percentage change in respiratory variables after LENART01 and ranatensin injection. The effect of LENART01 after blockade of MOR, BB1/BB2, and D2R and after cervical vagotomy. Baseline—pre-administration value, minimal (MIN) and maximal (MAX) percent changes in V_T_, F, and V_E_ within 2 min following drug injection. Box-plot format with median, Q1, Q3, min, max, and mean (x) values. * *p* < 0.05, ** *p* < 0.01 vs. the baseline value; #, o—*p* < 0.05; ##, oo—*p* < 0.01; #—vs. LENART01 group, o—vs. ranatensin group.

Intravenous administration of LENART01 resulted in a statistically significant reduction in tidal volume (V_T_) during a 2 min period post-injection, an elevation in respiratory rate (F) and due to alterations in V_T_ and F, a biphasic change in minute ventilation (V_E_), namely a decrease followed by a compensatory increase (Figure 1).

Following pre-treatment with sulpiride, no statistically significant effects of LENART01 on V_T_, F, and V_E_ were detected, though a trend towards decreased values in V_T_ and VE was noted. The administration of PD176252—an antagonist of BB1/BB2 receptors—as well as naloxone failed to affect effects mediated by the i.v. LENART01 challenge, although observed effects, especially after naloxone, were not as profound. Transection of the vagus nerve at the midcervical level abolished the respiratory response to the intravenous LENART01 challenge.

An equivalent dose of ranatensin, a component of the hybrid peptide under study, administered via the same route elicited a comparable respiratory response to LENART01, characterized by reduced V_T_, increased F, and biphasic changes in V_E_ (Figure 1); however, ranatensin did not provoke respiratory arrests.

**Figure 2:**
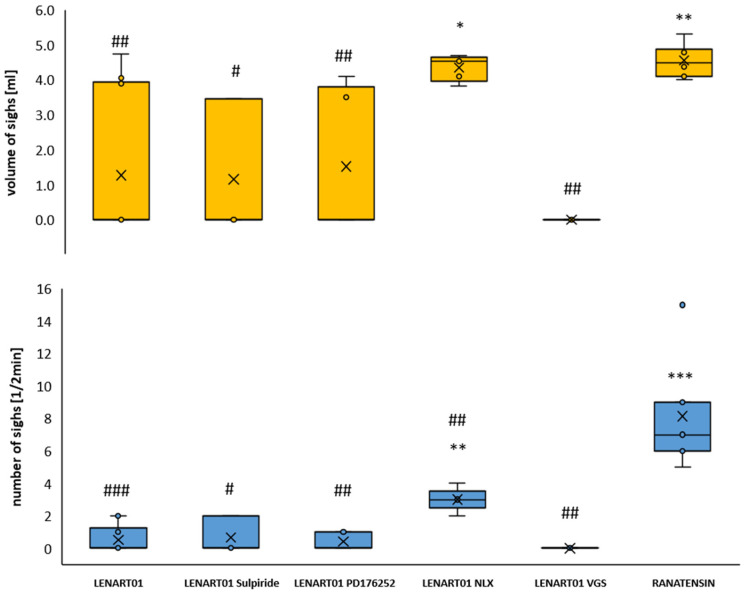
The number and the volume of sighs observed in the first 2 min following LENART01 and ranatensin challenge. The effect of LENART01 after blockade of MOR, BB1/BB2, and D2R and after cervical vagotomy. Note the increased volume and number of sighs after ranatensin injection, the lower number and even volume of sighs detected in the LENART01-treated animal groups, the increased number and volume of sighs after blockade of opioid receptors, and the lack of sighs after midcervical vagotomy. *, #—*p* < 0.05; **, ##—*p* < 0.01; ***, ###—*p* < 0.001; *—vs. LENART01; #—vs. ranatensin.

An additional observation was the presence of numerous sighs following administration of ranatensin in an equimolar dose to LENART01 (Figure 2). The sighs induced by ranatensin were of higher volume and also more numerous compared to all LENART01-treated groups. The number and volumes of all observed sighs were even among groups receiving LENART01 alone, after sulpiride, and after PD176252. The midcervical vagotomy abolished completely the appearance of sighs after the LENART01 challenge. The exception was the group in which MOR opioid receptors were inhibited by pre-treatment with naloxone, resulting in a significantly increased number and volume of sighs compared to the LENART01 group (Figure 2).

### 2.3. Cardiovascular Effects of LENART01: The Effects After Blockade of MOR, BB1/BB2, and D2R and After Cervical Vagotomy

**Figure 3:**
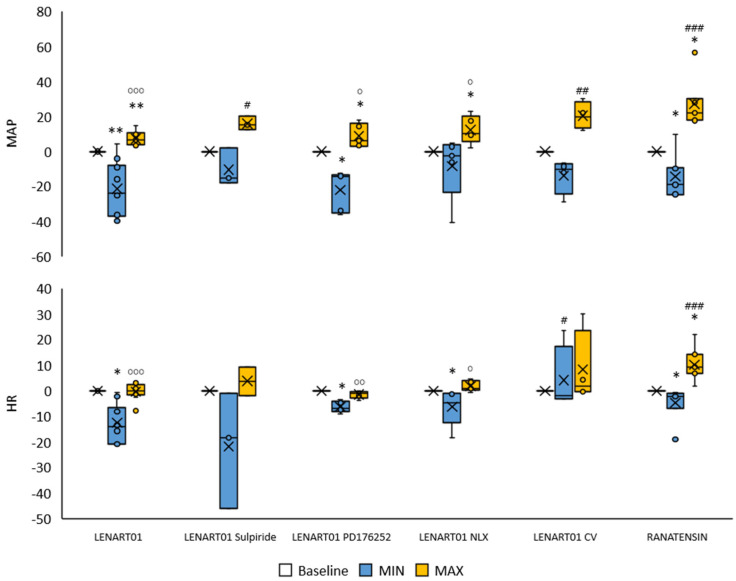
Percentage change in mean arterial blood pressure (MAP) and heart rate (HR) after LENART01 and ranatensin injection. The effect of LENART01 after blockade of MOR, BB1/BB2, and D2R and after cervical vagotomy. Baseline—pre-administration value, minimal (MIN) and maximal (MAX) percent changes in MAP and HR within 2 min following drug injection. Box-plot chart with median, Q1, Q3, min, max, and mean (x) value. #, o—*p* < 0.05; ##, oo—*p* < 0.01; ###, ooo—*p* < 0.001; # indicates the difference vs. the LENART01 group, and o indicates the difference vs. the ranatensin group, *—*p* < 0.05; **—*p* < 0.01 vs. baseline value.

Mean arterial blood pressure (MAP) showed a biphasic response after intravenous injection of LENART01, consisting of an initial decrease with a subsequent increase. The effect on heart rate (HR) was to slow it down briefly. The effect of ranatensin mirrored that of LENART01, with the exception of the effect on HR, which, after an initial slowdown, significantly increased (Figure 3). Use of inhibitors was not completely effective in blocking cardiovascular responses induced by LENART01. The changes in MAP and HR were significant after pre-treatment with PD176252 and naloxone, whereas changes after pre-treatment with sulpiride or subsequent to vagal dissection presented no statistical significance. Nevertheless, the trend in responses was similar, that is, a decrease in MAP with a subsequent increase and a decrease in HR. The exception was the response of HR after vagotomy, which eliminated its reduction after LENART01.

### 2.4. Comparison of the Respiratory Response Mediated by LENART01 with Its Pharmacophores Ranatensin and Dermorphin

**Figure 4:**
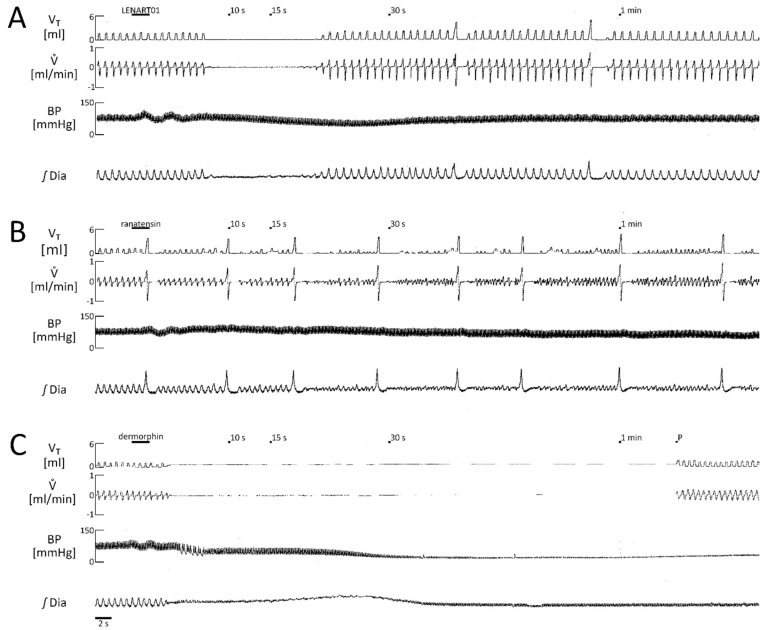
Representative recordings depicting tidal volume (V_T_), airflow (Vͦ), blood pressure (BP), and integrated electromyogram of the diaphragm (ꭍDia) in response to LENART01 (**A**), ranatensin (**B**), and dermophin (**C**) challenges. V.

Figure 4 shows representative recordings of tidal volume, flow, blood pressure, and diaphragm responses to LENART01, ranatensin, and dermorphin i.v. challenge. LENART01, as described above, induced apneas in 70% of the animals, 60% of which had apneas that terminated spontaneously (Figure 4A). An increase in tidal volume and sparse sighs were observed in returning respiration.

Ranatensin, one of the components of the hybrid, when injected i.v. at an equimolar dose to LENART01, did not cause apnea in any of the animals tested. Its main effect was frequent sighs with reduced V_T_ in between (Figure 4B). Nevertheless, in the case of dermorphin, an opioid component of LENART01, administered in a similar manner at an equimolar dose, abrupt arrest of breathing and a rapid decline in blood pressure were reported. Noteworthy, these effects were fatal to every animal out of five subjected to this treatment (Figure 4C).

## 3. Discussion

To the best of our knowledge, this is the first study to investigate the effects of ranatensin and its derived chimeric peptide LENART01 on the cardiovascular and respiratory systems.

Intravenous administration of LENART01 produced profound respiratory effects, including apnea in approximately 70% of the animals. Among the remaining rats, a biphasic ventilatory response was observed—initial respiratory depression followed by a compensatory increase in minute ventilation. The MAP response consisted of an initial decrease followed by an increase, while HR briefly slowed. Notably, aside from apnea, these responses are reminiscent of the respiratory and cardiovascular effects of ranatensin, which—until now—has not been studied in this physiological context.

In addition, a constant respiratory effect of ranatensin was the presence of sighs, which were also observed after administration of LENART01, but at a much lower frequency. Our hypothesis that ranatensin, due to its structural homology to bombesin [19,20], would exert similar respiratory effects was confirmed. Indeed, centrally administered bombesin has been reported to increase minute ventilation and induce periodic sighs [21]. Similarly, peripheral administration of bombesin was associated with sighing breathing, augmented tidal volume, decreased breathing frequency, and increased HR and mean arterial blood pressure. All respiratory and blood pressure responses were dependent on BB2 receptor activation [22]. Ranatensin’s cardiovascular effects were likewise comparable to those of bombesin, producing a consistent increase in blood pressure and HR [23]. Previously described effects of ranatensin on blood pressure varied depending on species and the level of baseline blood pressure [9]. It raised blood pressure in the dog and rabbit, lowered it in the monkey, had no influence in cats, and had variable action in guinea pigs. In rats, the variable effect was related to baseline blood pressure levels; in animals with high baseline blood pressure, it induced a hypotensive response; in those with low pressure, it induced a hypertensive response [9].

The respiratory and cardiovascular effects of dermorphin have already been described. While dermorphin is known primarily for its antinociceptive properties, it also modulates respiration. It has been shown that intracerebroventricular (i.c.v.) administration at lower doses in conscious rats increases ventilation and respiratory rate, whereas at higher doses the prevailing pattern is decreased ventilation by reducing the respiratory rate and delaying the increase in tidal volume [24,25]. In anesthetized rats, a consistent effect after both i.c.v. and i.v. administration of dermorphin was apnea [15,16,26] accompanied by a decrease in mean arterial blood pressure.

In contrast, although LENART01 does not cross the blood–brain barrier (BBB) [3,27], it nevertheless induced apnea in the majority of animals. This, along with the vagotomy effect preventing apnea, indicates that its action on the respiratory system is peripheral. Indeed, μ-opioid receptors are expressed not only centrally but also peripherally, particularly on vagal afferents, within the carotid body, and in circumventricular organs lacking a functional BBB, such as the area postrema. Activation of these sites can elicit potent respiratory depression, including apnea, without central nervous system entry [28,29].

Combining different mechanisms of action in a single molecule, leading to hybrid drugs, aims to produce more potent or novel biological effects that can improve their efficacy while facilitating treatment patterns. In our case, the pharmacophore fusion of ranatensin and dermorphin mitigated the adverse effects of both compounds on breathing, resulting in a milder side effect profile of the hybrid by reducing the number and intensity of apneas and decreasing substantially the number of sighs. As a reminder, LENART01 induced apnea in only 70% of animals, and most of them recovered spontaneously. Also, in regard to blood pressure response, instead of a major decrease caused by dermorphin and a significant increase caused by ranatensin, LENART01 induced a brief decrease in blood pressure and a subsequent increase but significantly lower than with ranatensin.

An additional aspect we wanted to explore was to determine the type of receptors that are involved in the specific respiratory and blood pressure responses of the hybrid. We took into consideration MOR, BB2, and D2 receptors, as dermorphin acts via MOR, ranatensin may act as homologous bombesin via BB2, and some studies indicate that ranatensin also has an affinity for D2R [12,30] and was able to induce D2R-dependent antinociception following i.c.v. administration in mice [11]. Neither the PD176252 antagonist of BB1/BB2R nor the sulpiride antagonist of D2R, which demonstrates not well-documented or limited ability to effectively cross the BBB or access cerebrospinal fluid [31,32], eliminated apnea, changes in breathing volume and rate, and sighs induced by LENART01 administration. This indicates that the BB2 and D2 receptor pathways are not essential for mediating the observed effects in this context. In contrast, naloxone completely abolished the apnea and significantly mitigated the reduction in minute ventilation and tidal volume. This finding points to a dominant role of MOR activation in the mediation of LENART01-induced respiratory depression. Again, considering that LENART01 does not cross the BBB [3], the blocking effect of naloxone does not necessarily reflect central opioid antagonism. Rather, it supports the notion that peripherally located MORs are sufficient to mediate this effect. Consequently, the overall results clearly suggest that the dominant mechanism responsible for its respiratory effects is peripheral μ-opioid receptor activation.

This is not particularly surprising, as the hybrid’s component dermorphin, a highly selective MOR agonist [33,34], has been shown to induce persistent apnea in anesthetized rats abolished by cutting the vagal pathway [16]. Consistent with this, we confirmed that midcervical vagotomy prevented apnea and attenuated other changes in respiratory parameters induced by LENART0. Interestingly, the sighs that also disappeared after cervical vagotomy were not blocked with naloxone, after which we even observed an increase in the number of sighs and their volume compared to the LENART01-treated group. This suggests that when MOR-mediated inhibition is blocked, the activity of the ranatensin moiety—presumably responsible for generating sighs—may become more prominent. Physiologically, sighs result from the summation of inspiratory drive from the vagus nerve and arterial chemoreceptors in the central nervous system [35]. While peripheral hypoxia can trigger augmented breaths via chemoreceptor activation, this mechanism requires facilitation by the vagus nerve [36]. Therefore, the presence or absence of sighs appears to reflect a functional integration between dopaminergic/bombesin-like activity and vagal afferent signaling, rather than a direct opioid receptor effect. Moreover, sighs may represent a compensatory mechanism to restore alveolar ventilation in response to reduced tidal volume induced by LENART01 or ranatensin alone.

Regarding the blood pressure response induced by LENART01, the initial lowering was eliminated by blocking MOR, D2R, and midcervical vagotomy, which is consistent with studies showing that activation of MOR with morphine or D2 receptors induces blood pressure lowering [37,38,39]. However, both the efficacy of naloxone blockade and vagotomy tend to point to the vagus nerve-related MOR receptor. In contrast, the subsequent rise in MAP continued despite the blockades or cutting of the vagus nerves, which indicates that the LENART01 pressor effect occurs beyond the vagal loop and is most probably mediated centrally. It cannot depend on central MOR receptors, as it was not blocked by naloxone hydrochloride, which passes through the blood–brain barrier (BBB) [40]. Thus, we cannot rule out the involvement of either central D2R or BB1/BB2 receptors in the generation of blood pressure increases induced by LENART01. In fact, in the central nervous system, postsynaptic D2R, are involved in increasing blood pressure [41]. We also showed in our previous study that homologous to ranatensin, bombesin evoked a similar MAP increase via most likely central BB2 receptors [22]. Thus, a limitation of our study is the lack of selective D2, BB1, and BB2 receptor antagonists that cross the BBB, which would clearly indicate their involvement in the hybrid’s pressure response.

Notably, another crucial limitation of our study is the absence of a group receiving a co-administration of dermorphin and ranatensin. While LENART01 was compared with each component individually, this design does not fully exclude additive pharmacodynamic effects. However, our intent was not to model polytherapy but to characterize the hybrid molecule itself. Further studies are needed to compare LENART01 directly with an equimolar mixture of its structural pharmacophores to elucidate hybrid-specific effects.

A limitation of the present study is also the lack of detailed pharmacokinetic and pharmacodynamic (PK/PD) analysis of the hybrid compound LENART01. As presented, the main objective of the work was to evaluate its physiological effects and to compare these effects with those of its individual pharmacophores—ranatensin derivative and dermorphin—in an experimental model that was not designed for a comprehensive determination of pharmacokinetic parameters such as bioavailability, half-life, volume of distribution, or metabolism. Although these aspects are important for the further therapeutic development of the compound and may influence the interpretation of its activity in vivo, they were not the subject of this analysis. Nevertheless, further studies aimed at a more comprehensive PK/PD characterization could provide additional context for the obtained results.

## 4. Materials and Methods

### 4.1. Animals

All animal procedures were approved by the Local Ethical Committee in Warsaw (permit No. WAW/097/2023, consent granted on 5 July 2023) and performed in accordance with the European Legislation (2010/63/EU). All experiments and methods were performed in accordance with relevant guidelines and regulations and are reported under the ARRIVE guidelines.

Experiments were performed on adult male Wistar rats (n = 22) weighing 258 ± 10 g (mean ± SD) under urethane (750 mg/kg) and α-chloralose (600 mg/kg; Sigma-Aldrich, Poznań, Poland) anesthesia administered intraperitoneally (i.p.). The level of unconsciousness was controlled by observing changes in blood pressure induced by pain-inducing stimuli (paw pinch). If necessary, additional doses of anesthesia were administered. The animals were placed in a supine position, and the rectal temperature was maintained close to 37–38 °C. The trachea was exposed, cut below the larynx, and attached to a tracheostomy tube, through which the animal breathed room air. The femoral artery for blood pressure monitoring and the femoral vein for intravenous (i.v.) drug administration were catheterized. Furthermore, in the group subjected to midcervical vagotomy, the vagus nerves, located adjacent to the left and right carotid arteries, were bluntly exposed and bilaterally transected below the superior laryngeal nerve branch.

### 4.2. Parameters’ Registration and Counting

A respiratory signal was registered with a pneumotachograph head connected to the tracheal cannula and linked to a research pneumotach recording system (RSS 100 HR, Hans Rudolph Inc., Kansas City, MO, USA), and respiratory variables such as tidal volume (V_T_), air flow, breathing frequency (F), minute ventilation (V_E_), time of inspiration (T_I_), and expiratory time (T_E_) were recorded. The electromyogram of the costal diaphragm was recorded from its sternal part with bipolar electrodes. The signal was amplified (1000–5000×) with an NL 104 amplifier (Digitimer Ltd., Hertfordshire, UK), band-pass filtered (50 Hz–50 kHz), and combined with a model AS 101 (Asbit, Parthenstein, Germany) leaky integrator (time constant = 100 ms). Arterial blood pressure and heart rate were measured with a BP-2 blood pressure monitor (Columbus Instruments, Columbus, OH, USA) connected to the catheter placed in the femoral artery.

The respiratory recordings (V_T_ and airflow (Vͦ)), blood pressure, and integrated electromyogram of the diaphragm were registered with an Omnilight 8 M 36 apparatus (Honeywell, Tokyo, Japan). The values of respiratory pattern parameters, mean arterial blood pressure (MAP), and heart rate (HR) were calculated by averaging the variables measured for five consecutive respiratory cycles just prior to drug injection (baseline) and at chosen time points of the post-challenge phase (5, 10, 15, 30, 60, 90, and 120 s after injection).

The maximal and minimal post-drug values in measured parameters were chosen from the computed time points ranging from the early post-drug phase to 2 min. The duration of the apneic period in expiratory airflow was measured as the time of respiratory arrest.

Prolongation of T_E_ was measured as the ratio of maximal T_E_ during post-drug apnea or expiration (T_E test_) to baseline expiratory time (T_E baseline_), T_E test_/T_E baseline_. The apneic pause longer than 30 s was terminated by application of a respiratory pump, and these registrations were excluded from further analysis.

### 4.3. Experimental Groups and Drugs

**Figure 5:**
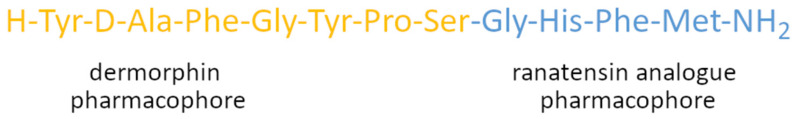
The amino acid sequence of LENART01 with the corresponding pharmacophores indicated.

LENART01 was synthesized as described elsewhere [13]. Its amino acid sequence is presented in Figure 5.

A stock solution of LENART01 was prepared weekly; it was dissolved in physiological saline and stored at −80 °C and refrigerated and dissolved to the final solution before the experiment. Sulpiride (Merck Life Science, Poznań, Poland)—a dopamine receptor antagonist, naloxone hydrochloride (Merck Life Science, Poznań, Poland)—an opioid receptor antagonist, and PD176252 (Bio-Techne, Warsaw, Poland)—bombesin BB1 and BB2 receptor antagonists were prepared freshly from powder before each experiment. Sulpiride was initially dissolved in a minimal volume (10 μL) of 10% aqueous HCl and brought to the final volume with NaCl; PD176252 in 150 μL of DMSO was administered i.p. The cardiovascular and respiratory variables were not altered by the administration of the respective vehicles (physiological saline or 10% HCl solution diluted in physiological saline for i.v. delivery and DMSO for i.p. delivery).

The experiments were conducted according to the following scheme:

1. LENART01 dose response (10 rats);
2. LENART01—naloxone, LENART01 (5 rats);
3. LENART01—sulpiride i.v., LENART01 (4 rats);
4. LENART01—cervical vagotomy, LENART01 (4 rats);
5. LENART01—PD176252 i.p., LENART01 (5 rats);
6. Ranatensin (5 rats);
7. Dermorphin (5 rats).

Since LENART01 did not show tachyphylaxis, while its half-life is short (data not published), it was administered more than once to each animal. Between the individual hybrid peptide injections, an interval of at least one hour was maintained.

Doses of the drugs were chosen based on the following: LENART01—preliminary dose response (see results, Table 1)—15 μg/kg (12 nmol/kg i.v.); naloxone—2 mg/kg i.v. [42]; sulpiride—25 mg/kg i.v. [43]; PD176252—4 mg/kg i.p. [32]; ranatensin and dermorphin—12 nmol/kg i.v. (dosage equimolar to the one selected for LENART01).

### 4.4. Statistics

Since not all the data were normally distributed and because of small group sizes, statistical analysis was carried out using non-parametric tests. The results are presented as medians with quartiles I and III and quartile deviations (QDs). A Wilcoxon test was used for comparison within the group between pre-challenge and defined time points after drug challenge. Differences between individual time points and groups were evaluated by the Mann–Whitney U test. The data were analyzed with STATISTICA 12 (StatSoft Polska, Kraków, Poland). In all cases, *p* ≤ 0.05 was considered statistically significant.

## 5. Conclusions

We have provided evidence that the effect of the dual opioid-dopamine receptor-related hybrid peptide, LENART01, on respiration and blood pressure response is a compilation of the actions of its pharmacophores and manifests itself in inducing apnea, sighing, and an overwhelming increase in blood pressure. At the same time, the hybrid induces sighs less frequently than ranatensin and apnea dependent on vagus nerve MOR receptor activation much less frequently and less intensely than dermorphin. This indicates that LENART01 is a safer alternative to activating MOR receptors than dermorphin for potential use in pain management.

## Figures and Tables

**Table 1:** Dose-dependent effect of LENART01 on apnea frequency, apnea duration, and expiratory time prolongation (T_E_/T_E baseline_ ratio).

LENART01 Dose	n	Number of Apnea Episodes Exceeding Duration of 30 s	Number of Apnea Episodes Shorter than 30 s	Apnea Duration [s] (Median (Q1, Q3) IQR)	T_E test_/T_E baseline_ (Median (Q1, Q3) IQR
5 μg/kg	5	0	0	0	1.0 (0.9, 1.0) 0.1 *
10 μg/kg	7	1	2	1.1	1.0 (0.9, 1.8) 1.0
**15** **μg/kg**	**7**	**1**	**3**	**6.1 (2.4, 12.2) 9.8**	**3.3 (1.0, 10.2) 9.2**
20 μg/kg	9	6	2	1.7	2.4 (0.8, 3.9) 3.1

* *p* < 0.05 indicates a statistical difference compared to the 15 μg/kg dose. Bold means the selected dose.

**Table 2:** Comparison of the effects of LENART01 on apnea incidence and tidal volume change before and after MOR (naloxone), BB1/BB2 (PD176252), and D2R (sulpiride) receptor blockade and after cervical vagotomy.

Treatment	LENART01	LENART01 Sulpiride (D2 R Antagonist)	LENART01 PD176252 (BB1/BB2 R Antagonist)	LENART01 Naloxone (MOR Antagonist)	LENART01 Cervical Vagotomy
**N**	14	4	5	5	4
**Number of apnea episodes exceeding duration of 30 s**	4	1	0	0	0
**Number of apnea episodes shorter than 30 s**	6	1	1	0	0
**Apnea duration [s] (median (Q1, Q3) IQR)**	11.2 (2.7, 13.5) 10.8	5.7	12.8	0	0
**T_E test_/T_E baseline_ (median(Q1, Q3) IQR**	3.8 (0.9, 20.9) 20.0	1.2 (0.9, 9.2) 8.3	0.8 (0.7, 16.5) 15.8	1.0 (0.9, 1.0) 0.1	1.0 (0.9, 1.0) 0.1
**Tidal volume (V_T_) [ml] (median (Q1, Q3) IQR) Baseline**	1.47 (1.15, 1.62) 0.47	1.31	1.42 (1.41, 1.62) 0.21	1.84 (1.56, 2.12) 0.54	2.02 (1.99, 2.15) 0.16
**Tidal volume at 10 s post injection (V_T_) [mL] (median (Q1, Q3) IQR)**	1.06 (0.54, 1.29) 0.75	1.02	1.17 (1.14, 1.23) 0.09	1.84 (1.51, 2.03) 0.52	2.05 (1.99, 2.12) 0.13
V_T_ **% of median change**	28	22	18	0	−1

## Data Availability

The data presented in this study are available in this article. The raw data of this study are available from the corresponding author upon request.
